# Case Report of Melody Valve Placement to Treat Neoaortic Valve Stenosis in an Adult With Fontan Circulation

**DOI:** 10.1016/j.jscai.2025.104018

**Published:** 2025-11-18

**Authors:** Matthew C. Schwartz, Jorge Alegria, Jonathan Schwartz, Joseph Paolillo

**Affiliations:** a Division of Pediatric Cardiology, Levine Children’s Hospital, Atrium Health, Charlotte, North Carolina; b Adult Congenital Heart Disease, Sanger Heart and Vascular Institute, Atrium Health, Charlotte, North Carolina; c Division of Adult Cardiology, Sanger Heart and Vascular Institute, Atrium Health, Charlotte, North Carolina

**Keywords:** case report, Fontan, Melody valve

## Abstract

The Melody valve (Medtronic) is commonly used for pulmonary valve replacement in patients with dysfunctional right ventricle to pulmonary artery conduits or bioprosthetic pulmonary valves. Rare reports exist of its use in the aortic position, including only 1 prior description of its use in a patient with hypoplastic left heart syndrome. We describe a 31-year-old patient with hypoplastic left heart syndrome who had severe neoaortic valve stenosis and moderate regurgitation that was successfully treated with percutaneous Melody valve placement in the neoaortic position.

## Introduction

One prior report describes the use of a transcatheter valve in a patient with hypoplastic left heart syndrome (HLHS) in the neoaortic position.^1^ We describe transcatheter placement of a Melody valve (Medtronic) in the neoaortic position in an adult male with HLHS to treat neoaortic valve stenosis and regurgitation.

## Case

The patient was born with critical aortic valve stenosis and underwent surgical valvotomy. The left ventricle was subsequently felt to be too small, and the patient underwent creation of Damus–Kaye–Stansil (DKS) anastomosis. At 9 months, the patient underwent right bidirectional Glenn operation and, at 3 years, underwent Fontan operation. He eventually developed severe neoaortic valve regurgitation and, at 23 years, underwent a complex operation that included takedown of the DKS anastomosis, replacement of the neoaortic root with a 20-mm Hemashield graft (Getinge), thinning of the neoaortic valve leaflets, and recreation of the DKS connection.

**Figure 1:**
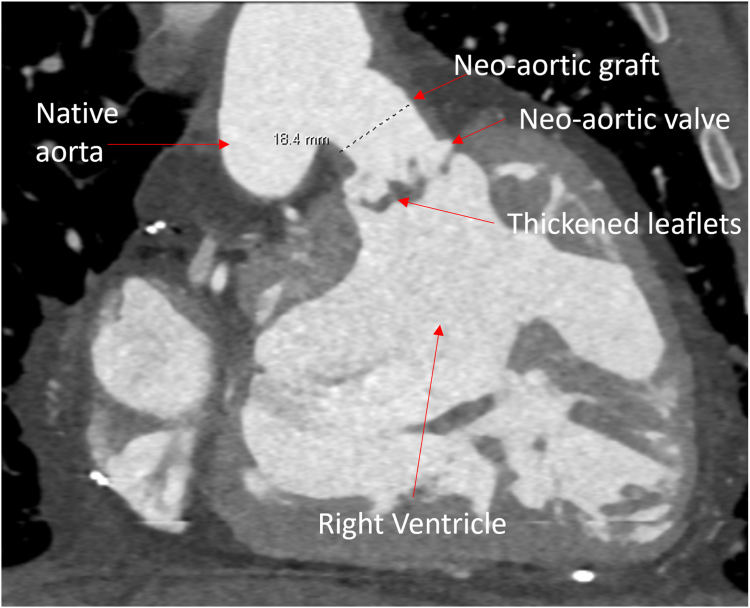
**Frontal projection from cardiac computed tomography showing the native aorta and its connection with the neoaortic hemashield graft.** The graft measures 18 mm in diameter. The neoaortic valve is seen with thickened leaflets.

At 31 years, the patient developed heart failure. Echocardiogram showed severe neoaortic valve stenosis with a peak instantaneous gradient of 84 mm Hg and mean of 63 mm Hg, as well as moderate regurgitation. The right ventricular (RV) systolic function was severely reduced. Cardiac computed tomography showed thickened neoaortic valve leaflets with limited systolic opening. The neoaortic graft measured 18 mm in the narrowest dimension (Figure 1). The patient was a poor surgical candidate. Transcatheter intervention was pursued as a bridge to possible eventual transplantation.

**Figure 2:**
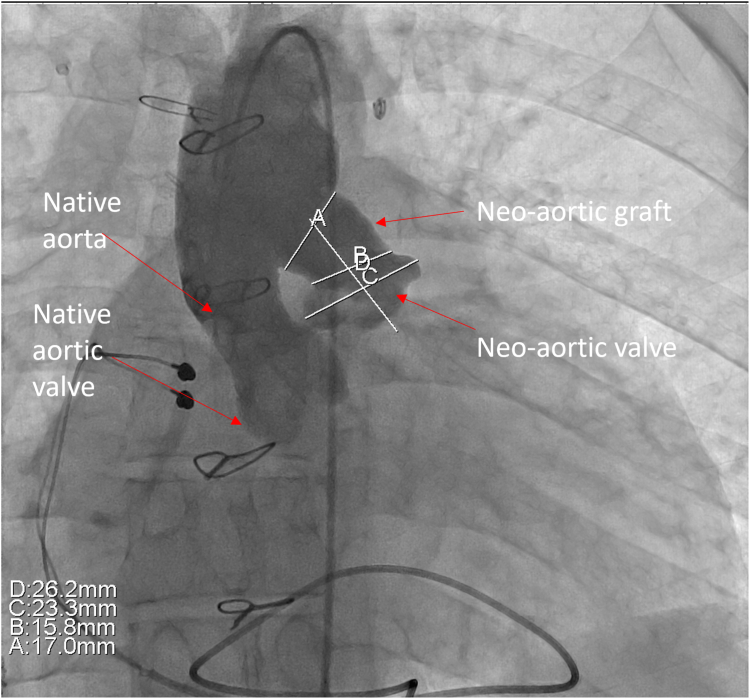
**Anteroposterior view of aortic angiogram showing the native aorta and its connection with the neoaortic graft**.

**Figure 3:**
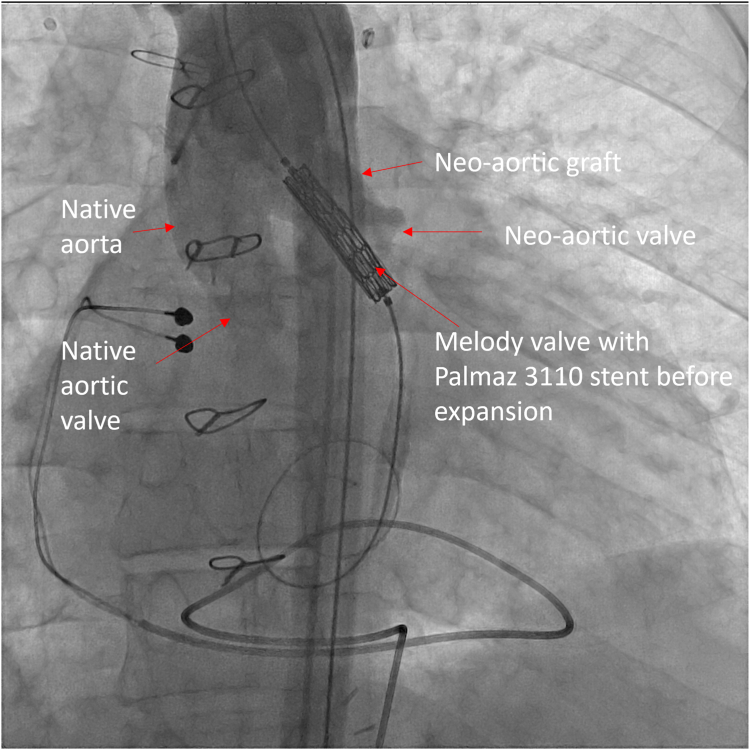
**Anteriorposterior view of aortic angiogram prior to expansion of the Melody valve.** A Melody valve and bare metal Palmaz stent have been crimped onto the 22-mm delivery ensemble, and the ensemble has been advanced retrograde over a Safari wire across the neoaortic valve. The valve is positioned to extend into the right ventricle to effectively pin or bridge the abnormal neoaortic valve leaflets. The valve will be anchored in the hemashield neoaortic graft but will not obstruct flow into the native aorta.

**Figure 4:**
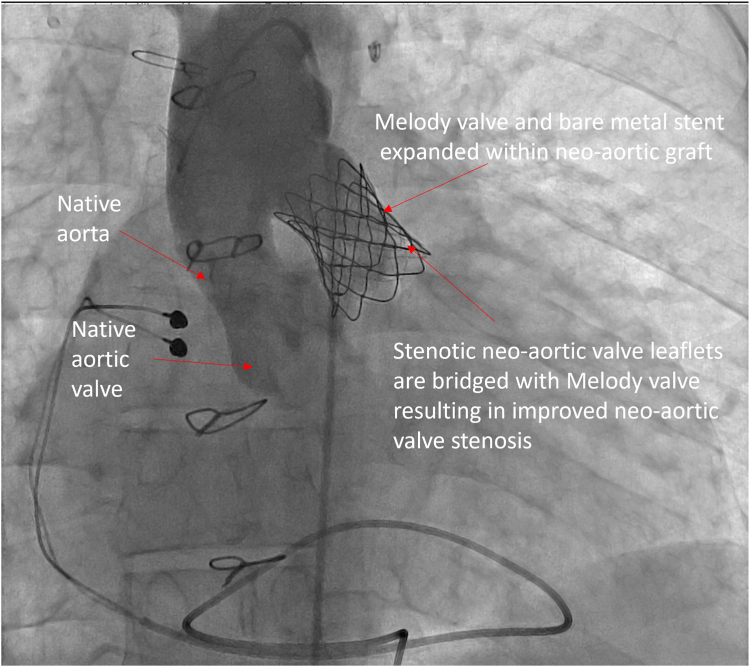
**Anteroposterior view of aortic angiogram after transcatheter replacement of the Melody valve in the neoaortic position showing that the stenotic neoaortic valve leaflets are bridged with the Melody valve**. There is no neoaortic valve regurgitation.

**Figure 5:**
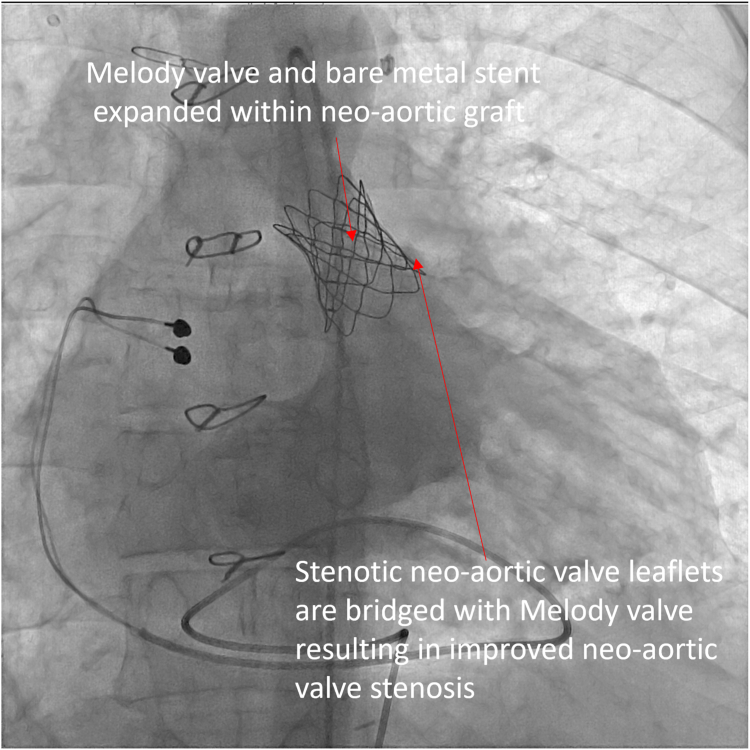
**Anteriorposterior view of a right ventricular angiogram after transcatheter replacement of the Melody valve in the neoaortic position showing that the stenotic neoaortic valve leaflets are bridged with the Melody valve.** The Melody valve is well-seated in the neoaortic graft.

Table 1 summarizes the hemodynamics at catheterization. Angiography showed that the neoaortic conduit narrowed to 15 mm. The valve leaflets were thick and were immediately proximal to the conduit (Figure 2; Supplemental Videos 1 and 2). The distance from the neoaortic valve annulus to the DKS connection was 25 mm. From the right femoral artery, the neoaortic valve was crossed retrograde and wire position established with a 0.035-inch Safari wire (Boston Scientific). The Melody valve was crimped onto a 22-mm Melody Ensemble delivery system. A Palmaz 3110 bare metal stent (Johnson & Johnson) was crimped on the outside of the valve. The ensemble was advanced retrograde over the Safari wire across the neoaortic valve. A pigtail catheter was advanced via the left femoral artery into the ascending aorta for angiography (Figure 3; Supplemental Video 3). RV pacing was performed via the Safari guide wire during valve expansion (Supplemental Video 4). After expansion, the gradient across the valve was improved, and there was no regurgitation (Table 1; Figures 4 and 5; Supplemental Videos 5 and 6).

**Table 1:** Variables measured at catheterization before and after neoaortic valve intervention.

Variables	Preintervention	Postintervention
Right ventricle pressure, mm Hg	190/20	131/15
Ascending aorta pressure, mm Hg	90/50	111/56
Mean Fontan conduit pressure, mm Hg	24	—
Mean pulmonary artery pressure, mm Hg	24	—
Pulmonary capillary wedge pressure, mm Hg	20	—
Cardiac index, L/min/m^2^	2.2	—
Pulmonary vascular resistance, WU	0.8	—

Table 2 describes the subsequent course. The patient’s heart failure symptoms significantly improved for 6 months, but at 1 year, the patient had moderate regurgitation and exertional symptoms. Transplant evaluation was pursued, but, unfortunately, 1 year after intervention, the patient died of septic shock associated with bacterial colitis.

**Table 2:** Echocardiographic, laboratory, and clinical variables before and after neoaortic valve intervention.

Variables	Before intervention	10 d after intervention	2 mo after intervention	8 mo after intervention	1 y after intervention
Neoaortic valve peak gradient by echocardiogram, mm Hg	84	35	38	36	42
Neoaortic valve mean gradient by echocardiogram, mm Hg	36	24	25	20	27
Neoaortic valve regurgitation by echocardiogram, mm Hg	Moderate	Trivial	Trivial	Mild to moderate	Moderate
B-type natriuretic peptide, pg/m	393	94	112	11	—
RV systolic function	Severely reduced	Mildly reduced	Normal	Normal	Mildly reduced
Symptoms	NYHA class III	NYHA class II	NYHA class II	NYHA class II	NYHA class II

NYHA, New York Heart Association; RV, right ventricular.

## Discussion

We describe the treatment of neoaortic valve dysfunction with transcatheter Melody valve placement in an adult with HLHS and a Fontan circulation. Martin et al^1^ previously reported transcatheter placement of a Melody valve in the neoaortic position in an 8-year-old patient with HLHS and Fontan circulation and severe neoaortic valve regurgitation. The Melody valve was placed in the neoaortic root via an antegrade approach from the right internal jugular vein across the Fontan conduit.^1^ Our patient had previously undergone neoaortic root replacement with a 20-mm hemashield graft and neoaortic valve repair. The graft acted as a landing zone for the transcatheter valve.

The Melody valve does have acceptable function in higher pressure positions with short-term follow-up. The patient reported by Martin et al^1^ had trivial Melody valve regurgitation at the 2-month follow-up. In addition, Hasan et al^2^ described placement of a Melody valve in 5 patients in the native aortic position and in 1 patient in a left ventricle to descending aorta conduit. Of these 6 patients, 2 had HLHS and a Fontan circulation and had Melody placement in the native aortic position for severe aortic regurgitation. In all these patients, none had more than mild regurgitation at a median follow-up of 2.9 months.^2^ Our patient had symptomatic improvement for 6 months and acceptable valve function during that time. However, at 1 year, the patient had moderate regurgitation with mild to moderate stenosis. It is unclear how durable the Melody valve will be in this high-pressure environment at a medium-term follow-up.

We used the Melody valve instead of a SAPIEN 3 valve (Edwards Lifesciences). The length of the Melody valve at a diameter of 20 mm is 24 mm. We considered that this length was optimal to not only help anchor the valve in the neoaortic graft but also extend proximal enough to “pin” the abnormal neoaortic valve leaflets and eliminate the valvar obstruction. The length of the 20-mm SAPIEN 3 is 15.5 mm; we considered it would be challenging to anchor the SAPIEN 3 in the conduit, while also pinning the neoaortic valve leaflets. Moreover, to ensure adequate expansion of the conduit, we crimped a Palmaz 3110 stent on the outside of the Melody valve as previously described for intervention in the pulmonary position.^3^^,^^4^ Alternatively, we could have crimped a bare metal stent on the outside of the SAPIEN 3 valve as the longer bare metal stent would have provided enough length to pin the neoaortic valve leaflets. The SAPIEN 3 likely would have been more durable than the Melody valve in this position. We chose not to pursue this strategy because we were concerned about dysfunction of the 20-mm SAPIEN 3 if the conduit were underexpanded to only 18 to 19 mm.

For RV pacing during valve expansion, we used a 0.035-inch Safari wire as the wire rail for Melody valve delivery and paced the ventricle using the end of this wire. This approach was simpler than transbaffle puncture and is well described to facilitate transcatheter aortic valve replacement.^5^

In conclusion, we describe replacement of a Melody valve in the neoaortic position in an adult with HLHS and a Fontan circulation who was a poor candidate for surgical valve replacement. He experienced short-term improvement, but, at 1 year, the Melody valve had moderate regurgitation and mild to moderate stenosis.
