# Supplementary material for: Case Report of Melody Valve Placement to Treat Neoaortic Valve Stenosis in an Adult With Fontan Circulation
Source: J Soc Cardiovasc Angiogr Interv. 2025 Nov 18;4(12):104018. doi: 10.1016/j.jscai.2025.104018 (PMC12766046; doi:10.1016/j.jscai.2025.104018)
Supplement: Supplemental Material [file mmc1.pptx]

## Slide 1
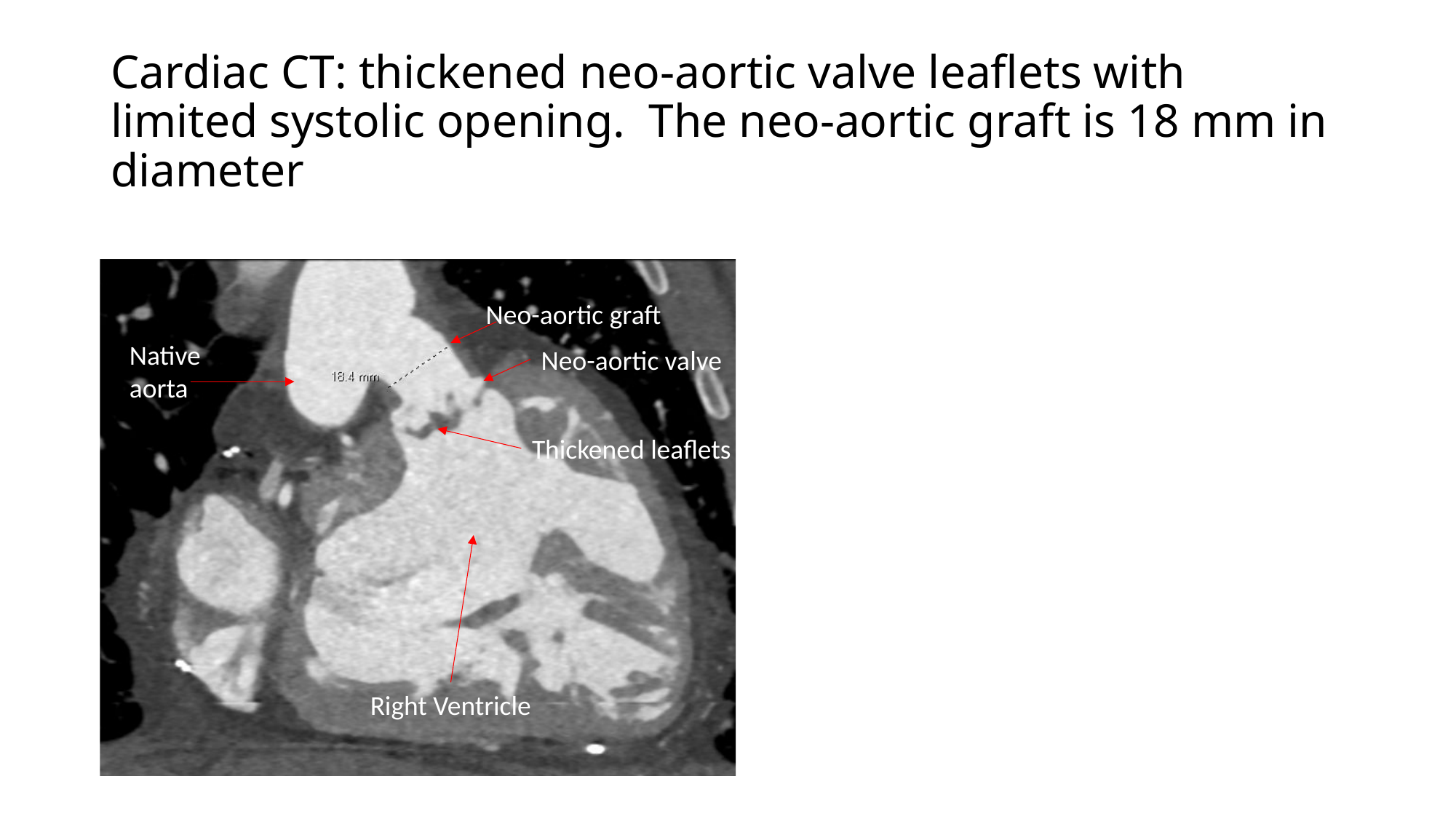

# Cardiac CT: thickened neo-aortic valve leaflets with limited systolic opening. The neo-aortic graft is 18 mm in diameter
Neo-aortic graft
Native
aorta
Neo-aortic valve
Thickened leaflets
Right Ventricle

## Slide 2
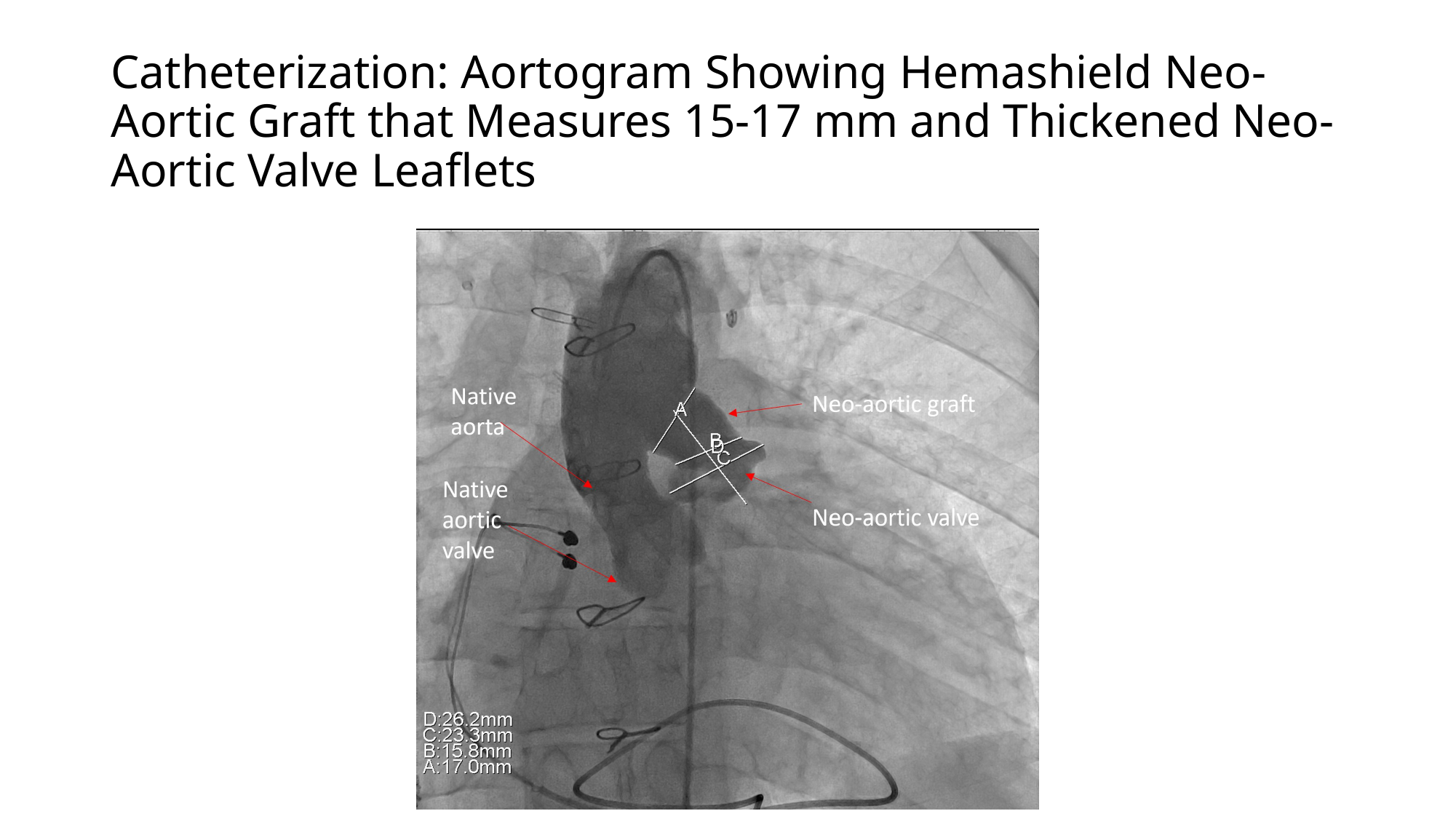

# Catheterization: Aortogram Showing Hemashield Neo-Aortic Graft that Measures 15-17 mm and Thickened Neo-Aortic Valve Leaflets

## Slide 3
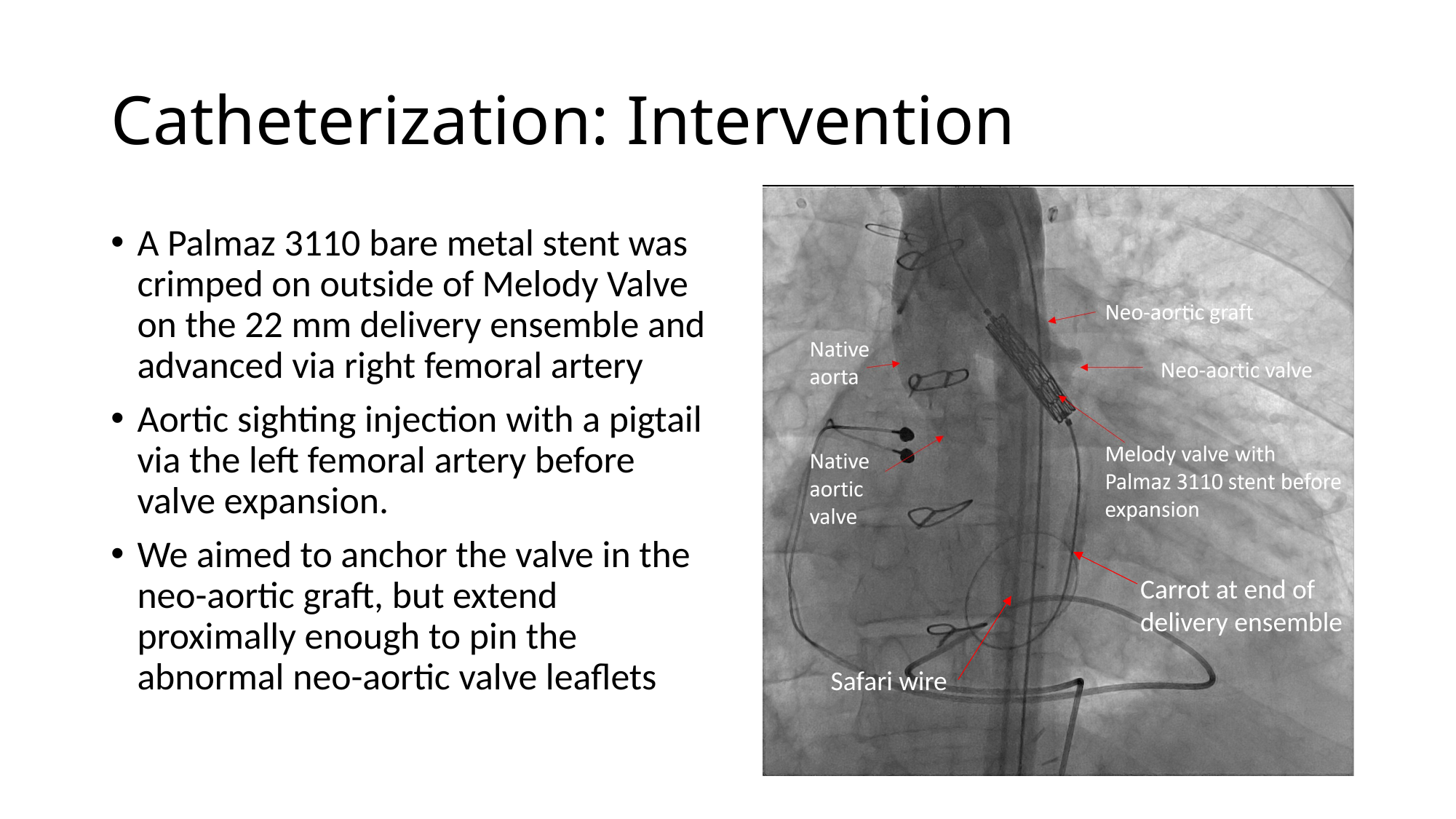

# Catheterization: Intervention
A Palmaz 3110 bare metal stent was crimped on outside of Melody Valve on the 22 mm delivery ensemble and advanced via right femoral artery
Aortic sighting injection with a pigtail via the left femoral artery before valve expansion.
We aimed to anchor the valve in the neo-aortic graft, but extend proximally enough to pin the abnormal neo-aortic valve leaflets
Carrot at end of
delivery ensemble
Safari wire

## Slide 4
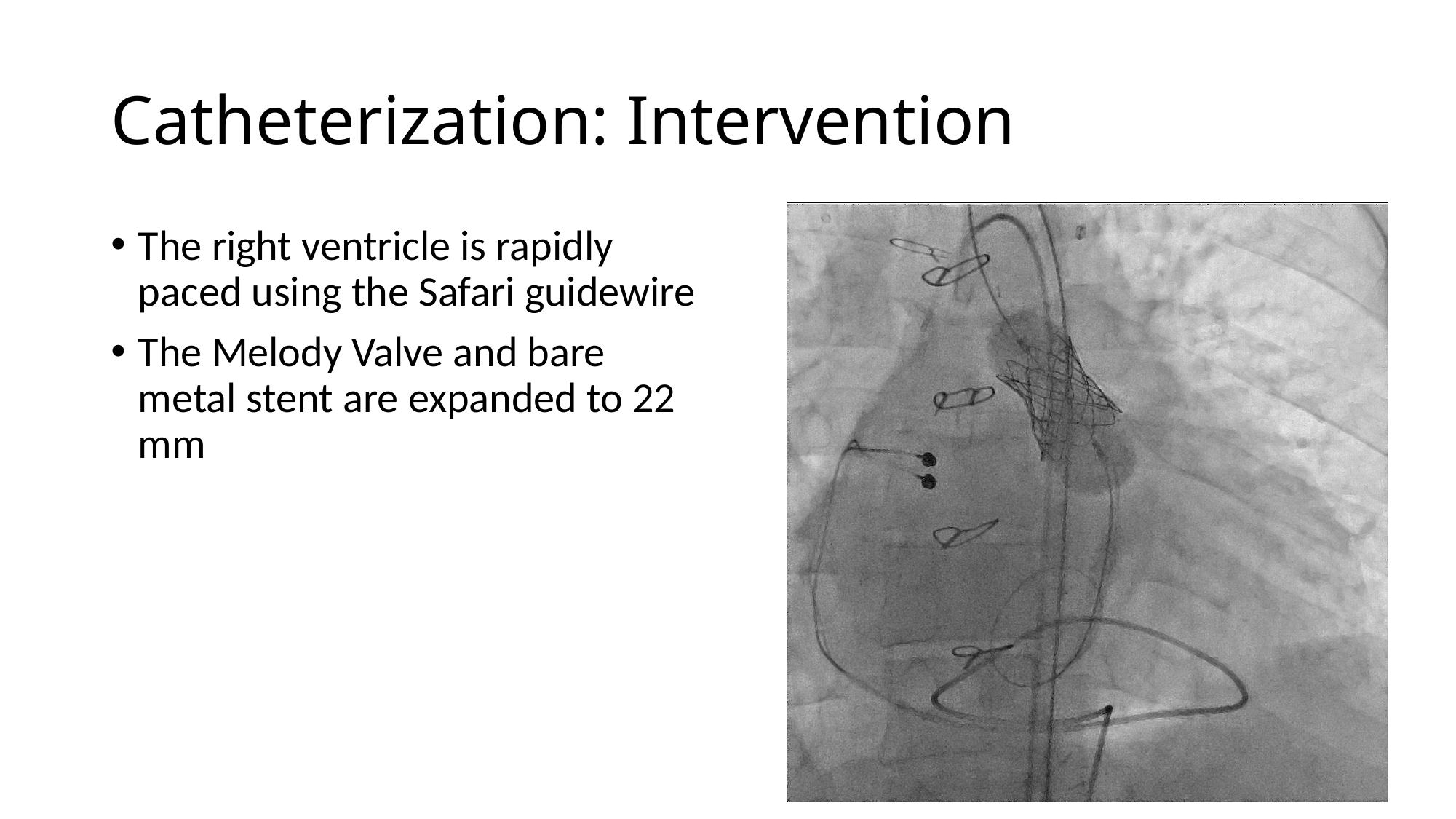

# Catheterization: Intervention
The right ventricle is rapidly paced using the Safari guidewire
The Melody Valve and bare metal stent are expanded to 22 mm

## Slide 5
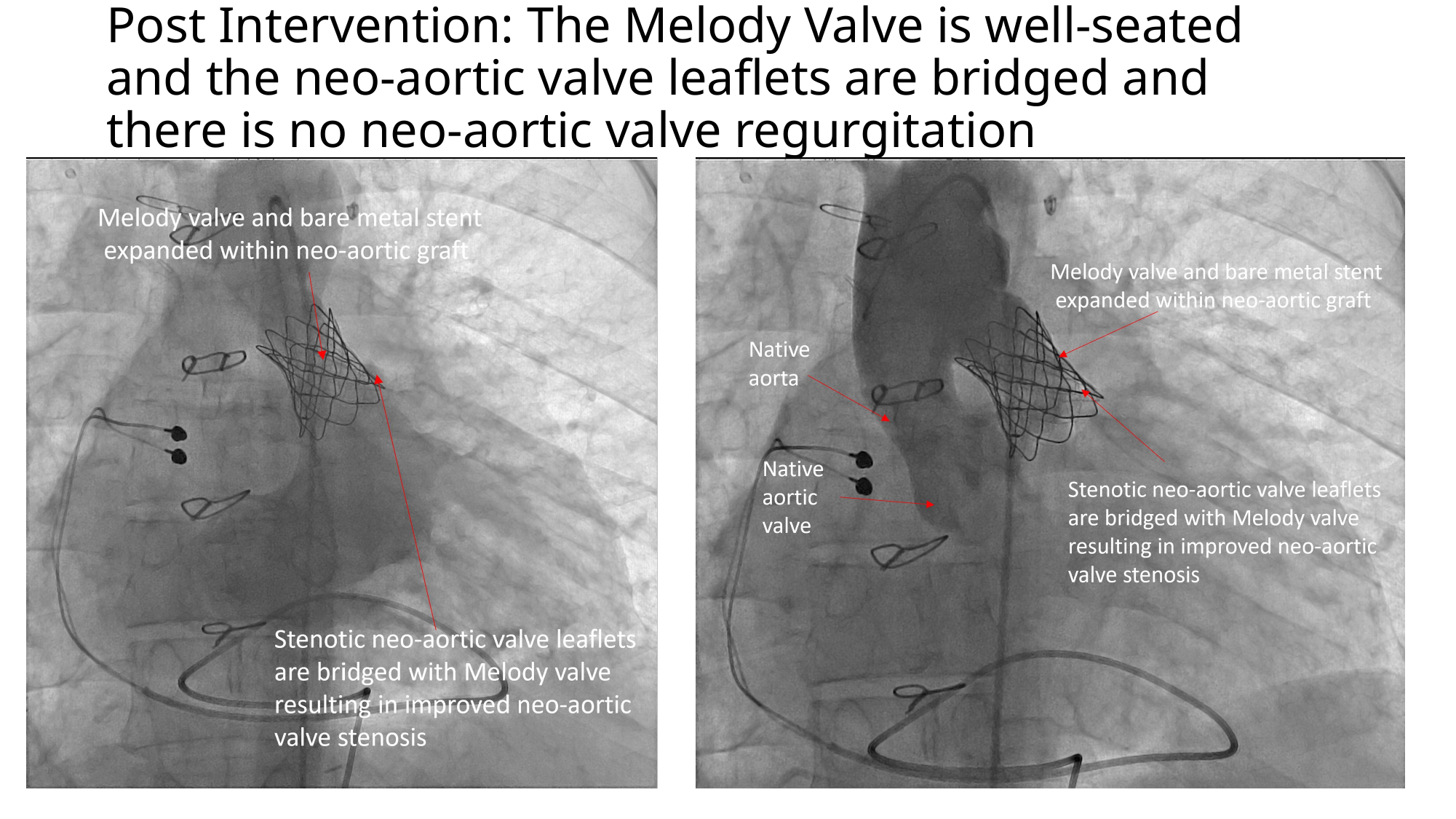

# Post Intervention: The Melody Valve is well-seated and the neo-aortic valve leaflets are bridged and there is no neo-aortic valve regurgitation
